# Pathogenic Mutation of TDP-43 Impairs RNA Processing in a Cell Type-Specific Manner: Implications for the Pathogenesis of ALS/FTLD

**DOI:** 10.1523/ENEURO.0061-22.2022

**Published:** 2022-06-07

**Authors:** Kent Imaizumi, Hirosato Ideno, Tsukika Sato, Satoru Morimoto, Hideyuki Okano

**Affiliations:** Department of Physiology, Keio University School of Medicine, Shinjuku, Tokyo 160-8582, Japan

**Keywords:** amyotrophic lateral sclerosis, frontotemporal lobar degeneration, induced pluripotent stem cell, TDP-43

## Abstract

Transactivating response element DNA-binding protein of 43 kDa (TDP-43), which is encoded by the *TARDBP* gene, is an RNA-binding protein with fundamental RNA processing activities, and its loss-of-function (LOF) has a central role in the pathogenesis of amyotrophic lateral sclerosis (ALS) and frontotemporal lobar degeneration (FTLD). *TARDBP* mutations are postulated to inactivate TDP-43 functions, leading to impaired RNA processing. However, it has not been fully examined how mutant TDP-43 affects global RNA regulation, especially in human cell models. Here, we examined global RNA processing in forebrain cortical neurons derived from human induced pluripotent stem cells (iPSCs) with a pathogenic *TARDBP* mutation encoding the TDP-43^K263E^ protein. In neurons expressing mutant TDP-43, we detected disrupted RNA regulation, including global changes in gene expression, missplicing, and aberrant polyadenylation, all of which were highly similar to those induced by TDP-43 knock-down. This mutation-induced TDP-43 LOF was not because of the cytoplasmic mislocalization of TDP-43. Intriguingly, in nonneuronal cells, including iPSCs and neural progenitor cells (NPCs), we did not observe impairments in RNA processing, thus indicating that the K263E mutation results in neuron-specific LOF of TDP-43. This study characterizes global RNA processing impairments induced by mutant TDP-43 and reveals the unprecedented cell type specificity of TDP-43 LOF in ALS/FTLD pathogenesis.

## Significance Statement

Altered RNA metabolism induced by transactivating response element DNA-binding protein of 43 kDa (TDP-43) loss-of-function (LOF) has been suggested to be a core disease mechanism in amyotrophic lateral sclerosis (ALS) and frontotemporal lobar degeneration (FTLD). Pathogenic mutations in *TARDBP* cause ALS and FTLD; thus, these mutations are considered to induce TDP-43 LOF. Here, using a human induced pluripotent stem cell (iPSC)-based model, we found that the pathogenic K263E mutation disrupted RNA processing events in neurons, reflecting TDP-43 LOF. Interestingly, in contrast to neuronal cultures, mutated TDP-43 remained functional in nonneuronal cells, including iPSCs and neural progenitors. This finding indicates an unprecedented neuron-specific LOF of TDP-43 that potentially accounts for the CNS-selective lesions in patients with ALS/FTLD.

## Introduction

Amyotrophic lateral sclerosis (ALS) is a neurodegenerative disorder caused by the degeneration of upper and lower motor neurons, leading to a progressive loss of motor function. Frontotemporal lobar degeneration (FTLD) is the second most common type of dementia after Alzheimer’s disease and is characterized by progressive atrophy in the frontal and temporal lobes and by personality and behavioral changes. Transactivating response element DNA-binding protein of 43 kDa (TDP-43) was identified as a major component of pathologic inclusions found in the vast majority of patients with ALS and ∼50% of patients with FTLD (Neumann et al., 2006). Moreover, previous studies reported mutations in the *TARDBP* gene encoding TDP-43 in patients with ALS and FTLD (Kabashi et al., 2008; Kovacs et al., 2009). These findings highlight a central role for TDP-43 in ALS/FTLD pathogenesis (S.C. Ling et al., 2013).

TDP-43 has various functions in RNA metabolism, including splicing, polyadenylation, transport, translation, and miRNA synthesis (S.C. Ling et al., 2013). Accumulating evidence suggests that TDP-43 loss-of-function (LOF) underlies the pathomechanism of ALS/FTLD. One particular target of TDP-43 LOF is RNA missplicing. TDP-43 LOF induces the cryptic splicing of a distinct group of genes, such as *STMN2*, which is also observed in patients with ALS/FTLD (Klim et al., 2019; Melamed et al., 2019; Prudencio et al., 2020). Notably, the pathogenesis of TDP-43 LOF is sometimes human-specific, requiring study in human cell models; for example, cryptic splicing of *STMN2* was not conserved in mouse models (J.P. Ling et al., 2015). Therefore, human induced pluripotent stem cell (iPSC)-based disease models have great potential (Okano and Morimoto, 2022). However, TDP-43 knock-down has been mainly used for the characterization of RNA processing impairments in human iPSC-based disease models (Klim et al., 2019; Prudencio et al., 2020), and the link between pathogenic *TARDBP* mutations and RNA regulation has not been fully explored. Another unanswered question is the cell type specificity of the effect of *TARDBP* mutations. As TDP-43 is globally expressed in various organs, including the brain, liver, lung, and kidney (Sephton et al., 2010), it is mysterious why these mutations result in ALS and FTLD, which selectively affect the CNS. Thus, the potential cell type specificity of the effect of the mutant TDP-43 protein on RNA regulation should be investigated.

In this study, using human iPSC-based models, we examined the K263E variant of TDP-43, which was initially identified in a patient with FTLD (Kovacs et al., 2009), with a disrupted RNA binding capacity (H.J. Chen et al., 2019). Similar to TDP-43 knock-down, TDP-43^K263E^ affected various RNA processing machineries in iPSC-derived neurons. These impaired RNA processes included intron splicing and 3′ polyadenylation regulation. In contrast to neurons, however, iPSCs and neural progenitor cells (NPCs) expressing TDP-43^K263E^ did not mimic TDP-43 LOF. This study characterizes global RNA processing impairments induced by mutant TDP-43 and indicates that mutant TDP-43 exhibits LOF in a cell type-specific manner.

## Materials and Methods

### Culture of undifferentiated iPSCs

Human iPSCs (201B7; Takahashi et al., 2007) were maintained in StemFit AK02 N medium (Ajinomoto). Cells were seeded at a density of 1.5 × 10^4^ cells/well in an iMatrix 511 (Laminin511E8; Wako)-treated six-well plate; 10 μm Y27632 (Nacalai) was only added for the first day. Culture media were changed every other day.

### Generation of TDP43^K263E^ isogenic iPSCs

CRISPR-Cas9-mediated homologous recombination was performed by GenAhead Bio. The sgRNA sequences targeting *TARDBP* were as follows: 5′-ATTGTGCTTAGGTTCGGCAT-3′and 5′-AATAGACAGTTAGAAAGAAG-3′. Both sequences were synthesized and ligated after the U6 promoter. The double-stranded targeting vector (TV) harboring the K263E mutation (c.A > G) together with the silent mutations at S258 (c.C > G) and S273 (c.A > T and c.G > C) flanked by 1-kbp homology arms was assembled using the NEBuilder HiFi DNA Assembly kit (New England BioLabs). iPSCs were electroporated with the Cas9 (D10A) expression vector, both sgRNA vectors, and TV at a ratio of 1:1:2 and verified as containing the biallelic K263E mutant using Sanger sequencing.

### Neuronal induction

Neuronal induction of iPSCs was performed using a previously described method (Sato et al., 2021), with slight modifications. Briefly, iPSCs were seeded on iMatrix511-coated 12-well plates at a density of 1.0–1.5 × 10^5^ cells/well in StemFit AK02 N medium. After 3 d, neural induction was initiated by changing the medium to neural induction medium [consisting of Advanced DMEM/F-12 (Thermo Fisher Scientific), 2% B27 supplement (–vitamin A; Thermo Fisher Scientific)] with 150 nm LDN193189 (StemRD), 5 μm SB431542 (Tocris), and 3 μm IWR1e (Calbiochem). On day 6, the cells were dissociated into single cells using Accutase (Nacalai) and seeded onto poly-L-ornithine-coated and laminin-coated 12-well plates at a 1:1–1:2 ratio. On day 12, the cells were dissociated again, and 8 × 105 cells seeded in each well of poly-L-ornithine-coated and laminin-coated six-well plates and cultured in neuronal medium [Advanced DMEM/F-12, 2% B27 supplement, 200 μm ascorbic acid (Sigma), and 200 μm dbcAMP (Sigma)] with 20 μm DAPT (Sigma). On day 18, DAPT was removed, and 10 ng/ml BDNF (Alomone Labs), 10 ng/ml GDNF (Alomone Labs), and 1 μm PD0332991 (Sigma) were added. The medium was changed every 3 d.

### RNA sequencing

Total RNA was isolated from iPSCs and NPCs on day 12 and from neurons on day 36 with the RNeasy Mini kit (QIAGEN) with DNase I treatment. The quality of RNA (RNA integrity number; RIN) was assessed by Agilent 2100 Bioanalyzer (Agilent). The indexed cDNA libraries were prepared using the TruSeq stranded mRNA Library Preparation kit (Illumina) and sequenced using a NovaSeq6000 (Illumina) to obtain 150-bp paired-end reads at Macrogen. RNA-seq datasets of TDP-43 knock-down experiments from previous reports Klim et al., 2019; Melamed et al., 2019) were downloaded via the NCBI Sequence Read Archive (accession numbers SRR8083864-8, SRR8083871-75, SRR8083878-81, and SRR8144907-12). The RNA-seq dataset of ESCs with TDP-43 knock-down (Modic et al., 2019) was kindly provided by M. Modic (Francis Crick Institute). Raw fastq files were trimmed to remove low-quality bases and adapters using fastp (S. Chen et al., 2018) and were processed for further analyses.

### Gene expression profiling

Salmon (Patro et al., 2017) was used to generate the TPM using the transcript index from the reference GRCh38 genome annotation (GENCODE release 33) to quantify gene expression levels. We identified differentially expressed genes (DEGs) using the DESeq2 suite of bioinformatics tools (Love et al., 2014) with a cutoff of 0.05 for Benjamini–Hochberg adjusted *p* values and a cutoff of 0.25 for the log2 fold change ratio. Principal component analysis (PCA) was performed using vst transformation of estimated counts based on intersections between DEG lists of two independent TDP-43 knock-down experiments from the studies by Klim et al. (2019) and Melamed et al. (2019). Our RNA-seq data were projected onto this PCA.

### Read alignment to the genome and alternative splicing analyses

HISAT2 (D. Kim et al., 2019) was used to map sequencing reads to the human GRCh38 genome. Coverage tracks were visualized with Integrative Genomics Viewer (IGV). Counts at individual exons were calculated from the HISAT2-aligned data using featureCounts (Liao et al., 2014) with the gene annotation from Ensembl (release 104), and the differential exon usage analysis was performed using DEXseq (Anders et al., 2012) with a cutoff of 0.01 for Benjamini–Hochberg adjusted *p* values. Differentially spliced intron clusters were analyzed from the HISAT2-aligned data without existing isoform annotations by LeafCutter (Li et al., 2018). Briefly, splice junction reads were extracted with RegTools (Cotto et al., 2021) using a minimum of 6 bp as an anchor on each side of the junction. Junctions from each sample were then clustered using leafcutter_cluster_regtools_py3.py (minclureads = 10). Differential intron splicing was calculated using leafcutter_ds.R. In addition to LeafCutter analysis, we also evaluated splice variants of *UNC13A* and *STMN2* by filtering reads that span the junctions between normal exons and cryptic exons as previously described (Ma et al., 2022). Junction spanning reads were filtered and quantified using junction_spanning_reads.sh.

### Alternative polyadenylation analysis

QAPA (Ha et al., 2018) was used to quantify TPM for individual alternative polyadenylation sites (PASs) from RNA-seq data. The 3′ untranslated region (UTR) sequence was extracted from GRCh38 genome by qapa fasta based on the precomplied annotation available on the QAPA GitHub page (https://github.com/morrislab/qapa). Salmon index was prepared using this 3′ UTR sequence. 3′ UTR isoform usage was then quantified using “salmon quant” and “qapa quant.” PASs with TPMs >5 were retained for further analysis. For each gene, gene-level relative PAS usage was summarized using a metric Ψ (Goering et al., 2021). Each PAS within a gene was assigned a value, *m*, which is defined as its position within this proximal-to-distal ordering, beginning with 1, to calculate Ψ. Each gene was also assigned a value, *n,* which is defined as the number of distinct PASs that it contains. The expression level of each PAS (*TPM_m_*) was evaluated, and gene-level PAS usage was summarized using the following formula:

$$\text{Ψ}=\frac{\sum_{m}\left(TPM_{m}\times \frac{m-1}{n-1}\right)}{\sum_{m}TPM_{m}}.$$

### Analysis of 3′ end-seq data

The 3′ end-seq datasets from a previous report (Modic et al., 2019) were downloaded from the European Nucleotide Archive (accession numbers ERR1642497 and ERR1642501). Fastp was used for quality and adapter trimming, and sequencing reads were aligned to the human GRCh38 genome using HISAT2. Coverage tracks were visualized using IGV.

### Quantitative RT-PCR

cDNA was prepared by using a ReverTraAce qPCR RT kit (Toyobo). The qPCR analysis was performed with TB Green Premix Ex Taq (TAKARA) using a ViiA 7 real-time PCR system (Applied Biosystems) according to the manufacturer’s instructions. Values were normalized to *ACTB* levels. Data were analyzed using the comparative (ΔΔCt) method. The primers used for qPCR were as follows: *ACTB*, forward 5′-TGAAGTGTGACGTGGACATC-3′, reverse 5′-GGAGGAGCAATGATCTTGAT-3′; *FOXG1*, forward 5′-CCCGTCAATGACTTCGCAGA-3′, reverse 5′-GTCCCGTCGTAAAACTTGGC-3′; *OCT4*, forward 5′-GACAGGGGGAGGGGAGGAGCTAGG-3′, reverse 5′-CTTCCCTCCAACCAGTTGCCCCAAAC-3′; *PAX6,* forward 5′-ACCACACCGGTTTCCTCCTTCACA-3′, reverse 5′-TTGCCATGGTGAAGCTGGGCAT-3′; *TARDBP-*Δ*Intron7*, forward 5′-TTCATCTCATTTCAAATGTTTATGGAAG-3′, reverse 5′- ATTAACTGCTATGAATTCTTTGCATTCAG-3′; *TUBB3,* forward 5′-ATTTCATCTTTGGTCAGAGTGGGGC-3′, reverse 5′-TGCAGGCAGTCGCAGTTTTCAC-3′; and *UNC13A-CE*, forward 5′-TGGATGGAGAGATGGAACCT-3′, reverse 5′-GGGCTGTCTCATCGTAGTAAAC-3′.

### Immunocytochemistry

Neurons cultured until day 36 were fixed with 4% paraformaldehyde for 15 min at room temperature and then washed three times with PBS. After an incubation with blocking buffer (PBS containing 5% normal goat serum and 0.3% Triton X-100) for 30 min at room temperature, the cells were incubated overnight at 4°C with primary antibodies at the following dilutions: TDP-43 (rabbit, Proteintech, 10782-2-AP, 1:200) and TUBB3 (mouse, Sigma, T8660, 1:500). The cells were again washed three times with PBS and incubated with secondary antibodies conjugated to Alexa Fluor 488 or 555 (Life Technologies) and Hoechst 33342 (Dojindo Laboratories) for 1 h at room temperature. After three washes with PBS and one wash with distilled water, the samples were mounted on slides and examined using an LSM-710 confocal laser scanning microscope (Carl Zeiss). Line-scan analysis was performed using ImageJ software. The resulting values were normalized to the maximum intensity. For the analysis of TDP-43 localization, TUBB3 staining was used to determine the cell body as the region of interest, and then Pearson’s correlation coefficient was calculated for TDP-43 and Hoechst staining using the Coloc2 plugin.

### Data availability

All the sequencing data have been deposited in the NCBI’s Gene Expression Omnibus and are accessible through GEO Series accession number GSE195689 (201B7 iPSC) and GSE196144 (otherwise).

## Results

### Gene expression profile of cortical neurons derived from isogenic iPSCs harboring the TDP-43 K263E mutation

**Figure 1.**
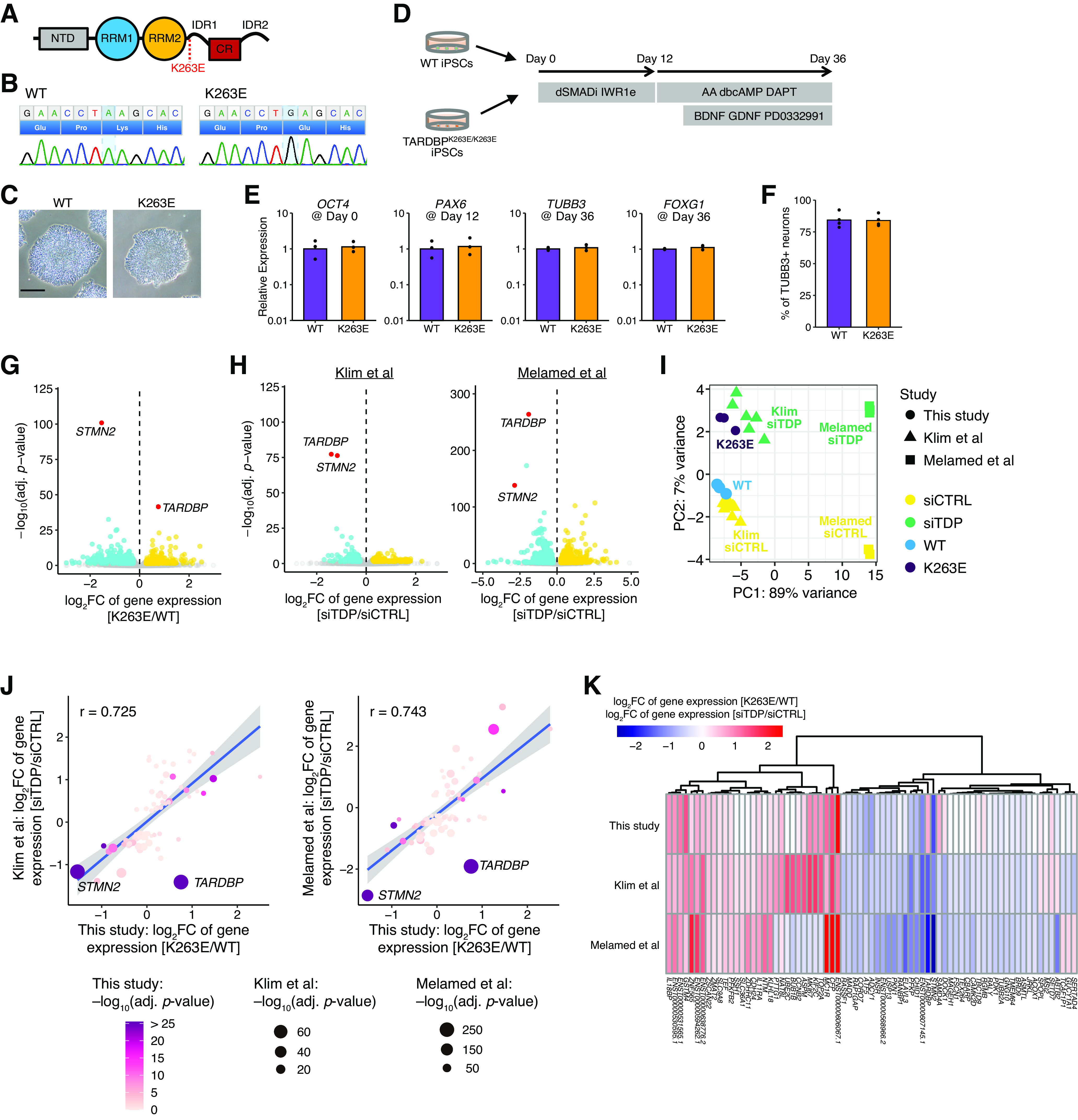
K263E mutation and TDP-43 knock-down exert similar effects on gene expression. ***A***, The domain map of TDP-43. The position of the K263E mutation is shown. NTD, N-terminal domain; RPM, RNA recognition motifs; IDR, intrinsically disordered regions; CR, conserved region. ***B***, Genomic DNA sequencing of *TARDBP* isogenic iPSC lines. ***C***, Bright-field images of wild-type and *TARDBP* isogenic iPSCs. Scale bar: 200 μm. ***D***, Schematic of the differentiation of iPSCs into cortical neurons. ***E***, Fold changes in the expression of *OCT4* in iPSC, *PAX6* in NPC, and *TUBB3*/*FOXG1* in neuron cultures (normalized to *ACTB*; *n* = 3). ***F***, Quantification of the number of TUBB3+ neurons at day 36 (*n* = 4). ***G***, Volcano plot showing genes with significantly altered expression in cortical neurons derived from TDP-43^K263E^ iPSCs relative to those from wild-type iPSCs. DEGs were identified using the Wald test with a cutoff of 0.05 for Benjamini–Hochberg adjusted *p* values and a cutoff of 0.25 for the log2 fold change ratio. Quality control of RNA-seq data are shown in Extended Data Figure 1-1. ***H***, Volcano plots showing genes with significantly altered expression following TDP-43 knock-down in iPSC-derived neurons and SH-SY5Y cells. The published RNA-seq datasets (Klim et al., 2019; Melamed et al., 2019) were reanalyzed. ***I***, PCA of RNA-seq datasets based on DEGs induced by TDP-43 knock-down. ***J***, ***K***, Scatter plot (***J***) and heatmap (***K***) comparing the fold changes in gene expression induced by TDP-43^K263E^ and TDP-43 knock-down. Analyzed genes were selected as intersections between the DEG lists of two independent TDP-43 knock-down experiments from studies by Klim and colleagues and Melamed and colleagues without additional filters. Shaded areas indicate 95% confidence intervals. Pearson’s correlation coefficients were calculated after excluding the *TARDBP* values.

In the present study, we focused on the function of the mutant TDP-43^K263E^ protein (Fig. 1*A*). This mutation of the *TARDBP* gene was identified in a patient with FTLD (Kovacs et al., 2009) and is suggested to reduce the RNA binding capacity of TDP-43 (H.J. Chen et al., 2019). We introduced a homozygous K263E mutation in healthy wild-type human iPSCs using the CRISPR/Cas9 system (Fig. 1*B*,*C*). Forebrain cortical neurons were generated from control (wild-type) and TDP-43^K263E^ iPSCs by dual SMAD and Wnt inhibition, respectively (Fig. 1*D*; Imaizumi et al., 2015; Sato et al., 2021). This mutant genotype did not affect the gene expression of markers for pluripotency, neural progenitors, and neurons (Fig. 1*E*). In addition, we found no difference in neuronal induction efficiency (Fig. 1*F*). These data indicate that the pluripotency maintenance and the neuronal induction were equivalent between wild-type and TDP-43^K263E^ iPSCs.

10.1523/ENEURO.0061-22.2022.f1-1 Extended Data Figure 1-1 Quality of RNA-seq data. ***A***, RIN of RNA samples subjected to sequencing. ***B***, Average Phred score of prefiltered sequencing data per read position. ***C***, Filtering result by fastp. ***D***, ***E***, Mapping rate of alignment by HISAT2 (***D***) and pseudo-alignment by Salmon (***E***). ***F***, The variance of DEGs in neurons derived from TDP-43^K263E^ iPSCs relative to those from wild-type iPSCs. Download Figure 1-1, EPS file.

We performed an RNA-seq analysis of these iPSC-derived cortical neurons. Data quality was assured by RIN, Phred quality score, and a uniform mapping rate for all samples (Extended Data Fig. 1-1*A–E*). The global gene expression profile was measured, and the analysis of DEGs identified 550 genes that were significantly differentially expressed between wild-type and TDP-43^K263E^ iPSC-derived neurons (Fig. 1*G*; Extended Data Fig. 1-1*F*). Fold changes of these DEGs were relatively small, but this is consistent with other reports (Klim et al., 2019; Melamed et al., 2019). Among these DEGs, the most significantly altered gene was *STMN2*. As previous studies have reported that *STMN2* is the gene most affected by TDP-43 knock-down (Klim et al., 2019; Melamed et al., 2019), we reanalyzed RNA-seq datasets from these previous studies and compared the effect of TDP-43^K263E^ and TDP-43 knock-down on gene expression. TDP-43 knock-down downregulated *STMN2* expression quite similarly to TDP-43^K263E^ (Fig. 1*H*). PCA grouped TDP-43^K263E^ and TDP-43 knock-down cells into the same cluster along PC2, whereas the data from the study by Melamed and colleagues were grouped separately on PC1 (Fig. 1*I*). When comparing gene expression between TDP-43^K263E^ and TDP-43 knock-down neurons, a strong correlation was observed between TDP-43^K263E^ and TDP-43 knock-down neurons, except for the expression of *TARDBP* (Fig. 1*J*,*K*). Collectively, TDP-43^K263E^ and TDP-43 knock-down exerted similar effects on gene expression, indicating that K263E corresponds to an LOF mutation.

### Characterization of missplicing indicates the similarity between TDP-43^K263E^ and TDP-43 knock-down

**Figure 2.**
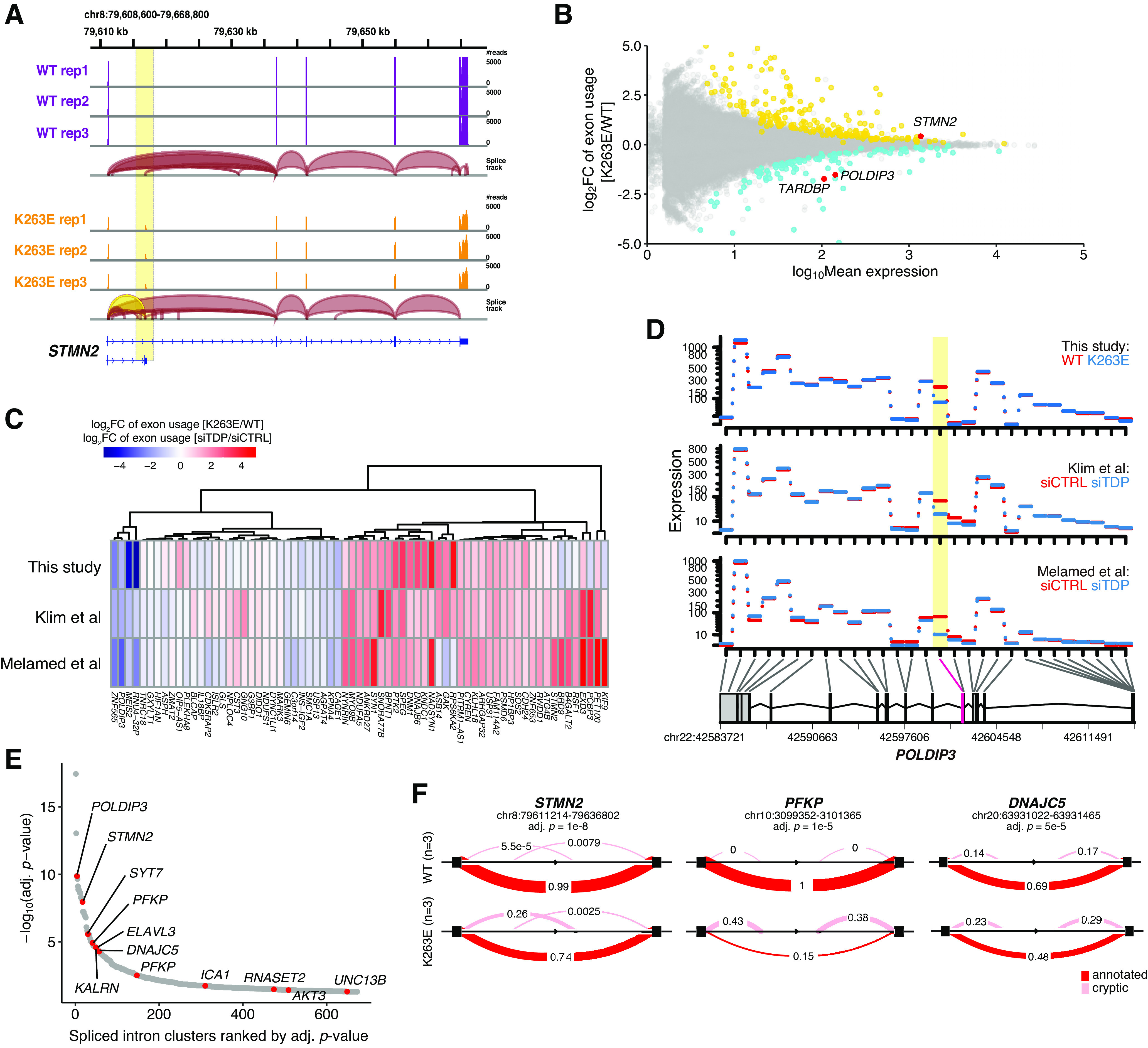
RNA splicing alterations induced by TDP-43^K263E^. ***A***, RNA-seq read coverage and splice junctions mapped to the genomic region of *STMN2*. The cryptic exon and splice ribbon from exon 1 to the cryptic exon are highlighted in yellow. ***B***, Scatter plot of differential exon usage between TDP-43^K263E^ and wild-type iPSC-derived cortical neurons. The expression level at individual exons was quantified. The exon with the most significantly altered expression in each gene is shown. Differentially used exons were identified with a cutoff of 0.01 for Benjamini–Hochberg adjusted *p* values. ***C***, Heatmap comparing fold changes in exon usage induced by TDP-43^K263E^ and TDP-43 knock-down. Analyzed genes were selected based on intersections between differentially used exon lists of two independent TDP-43 knock-down experiments from studies by Klim and colleagues and Melamed and colleagues. ***D***, Visualization of the expression levels of each of the exons of *POLDIP3*. Exons with significantly different usage levels are highlighted in yellow. ***E***, Differentially spliced intron clusters identified by LeafCutter analysis with a cutoff of 0.05 for Benjamini–Hochberg adjusted *p* values. Shown are the names of the genes to which the intron clusters belong. ***F***, Differentially spliced intron clusters in *STMN2*, *PFKP*, and *DNAJC5* are shown. Exons, black boxes. The thickness of the red band and inserted values represent the proportions of spliced pairs. The evaluation of *UNC13A* splicing is shown in Extended Data Figure 2-1.

TDP-43 plays a pivotal role in RNA processing, and previous reports suggest that *STMN2* loss on TDP-43 knock-down is because of cryptic exon inclusion (Klim et al., 2019; Melamed et al., 2019). Notably, we identified the same cryptic splice events in TDP-43^K263E^ neurons (Fig. 2*A*). This observation suggests that TDP-43^K263E^ induced RNA missplicing; therefore, we analyzed differential exon usage between wild-type and TDP-43^K263E^ neurons using RNA-seq datasets. We identified 299 genes whose exon usages were significantly altered (Fig. 2*B*). We repeated the same exon usage analyses for TDP-43 knock-down datasets, and fold changes in exon usage induced by TDP-43^K263E^ were highly similar to those induced by TDP-43 knock-down (Fig. 2*C*). Notably, both in TDP-43^K263E^ and in TDP-43 knock-down cells, we detected the exclusion of *POLDIP3* exon 3 (Fig. 2*D*), which has previously been associated with TDP-43 deficits (Shiga et al., 2012). Based on these data, RNA missplicing induced by TDP-43^K263E^ results from TDP-43 LOF.

10.1523/ENEURO.0061-22.2022.f2-1 Extended Data Figure 2-1 *UNC13A* splicing. ***A***, Schematic of alternative splicing of *UNC13A*. CE, cryptic exon. ***B***, Reads that span either exon 20–exon 21 junction, exon 20–CE junction, or CE–exon 21 junction were quantified. ***C***, Percentages of cryptic splicing of *UNC13A* based on junction spanning read counts. The numbers written above the graphs are the number of samples in which cryptic splicing was detected. ***D***, Schematic of alternative splicing of *STMN2*. ***E***, Reads that span either exon 1–exon 2 junction or exon 1–CE junction were quantified. ***F***, Percentages of cryptic splicing of *STMN2* based on junction spanning read counts. ***G***, Genomic DNA sequencing of risk SNPs in *UNC13A* intron. ***H***, RT-qPCR confirmed inclusion of CE in *UNC13A* mRNA in TDP-43^K263E^ iPSC-derived neurons (normalized to *ACTB*; *n* = 3). ***I***, Percentages of cryptic splicing of *UNC13A* based on junction spanning read counts in Klim et al. (2019) and Melamed et al. (2019). The numbers written above the graphs are the number of samples in which cryptic splicing was detected. ^#^One sample in Melamed et al. contained no reads spanning exon 20–exon 21 junction, and was excluded for the splicing rate quantification. Download Figure 2-1, EPS file.

The exon usage analysis is based on existing isoform annotations, but disease-relevant missplicing often occurs in unannotated introns. We performed an annotation-free differential splicing analysis to characterize global changes in intron splicing (Li et al., 2018). We detected cryptic splicing not only in *STMN2* but also in various gene targets, including *PFKP*, *DNAJC5*, *KALRN*, *SYT7*, and *UNC13B*, all of which have been shown to be misspliced in TDP-43 knock-down models (Fig. 2*E*,*F*; Polymenidou et al., 2011; Klim et al., 2019; Brown et al., 2022; Ma et al., 2022). On the other hand, this annotation-free splicing analysis could not detect *UNC13A* cryptic splicing, which has recently been reported as a hallmark of TDP-43 LOF (Brown et al., 2022; Ma et al., 2022). To further evaluate *UNC13A* splicing, sequencing reads spanning the cryptic exon were extracted and quantified. We detected very few spanning reads in only a subset of samples of TDP-43^K263E^ neurons, whereas there were no spanning reads in wild-type neurons (Extended Data Fig. 2-1*A–C*). A similar analysis focusing on *STMN2* cryptic exon robustly detected splicing change by mutant TDP-43 (Extended Data Fig. 2-1*D–F*). These results raised two possibilities: one is that TDP-43^K263E^ has little if any effect on *UNC13A* splicing; and the other is that RNA sequencing could not well capture *UNC13A* cryptic exons despite the fact that missplicing occurs. We found that both wild-type and isogenic TDP-43^K263E^ cells harbored homozygous risk SNP/indels (rs12973192, rs12608932, and rs56041637) that increase the efficiency of *UNC13A* cryptic splicing on TDP-43 loss (Extended Data Fig. 2-1*G*; Brown et al., 2022; Ma et al., 2022). Thus, the cryptic splicing of *UNC13A* in these cells is expected to be drastically enhanced by TDP-43 LOF, which supports the second possibility. Indeed, RT-qPCR analysis revealed that *UNC13A* cryptic splicing was reproducibly increased in TDP-43^K263E^ neurons (Extended Data Fig. 2-1*H*). These data suggest that *UNC13A* missplicing was actually occurring, but RNA-seq analysis was not sensitive enough to detect it. The low sensitivity of RNA-seq analysis to detect *UNC13A* missplicing was also supported by the finding that analyses using the data from Klim et al. (2019) and Melamed et al. (2019) similarly detected *UNC13A* missplicing in only a subset of samples (Extended Data Fig. 2-1*I*).

### TDP-43 autoregulation through RNA 3′ end processing is impaired by TDP-43^K263E^

**Figure 3.**
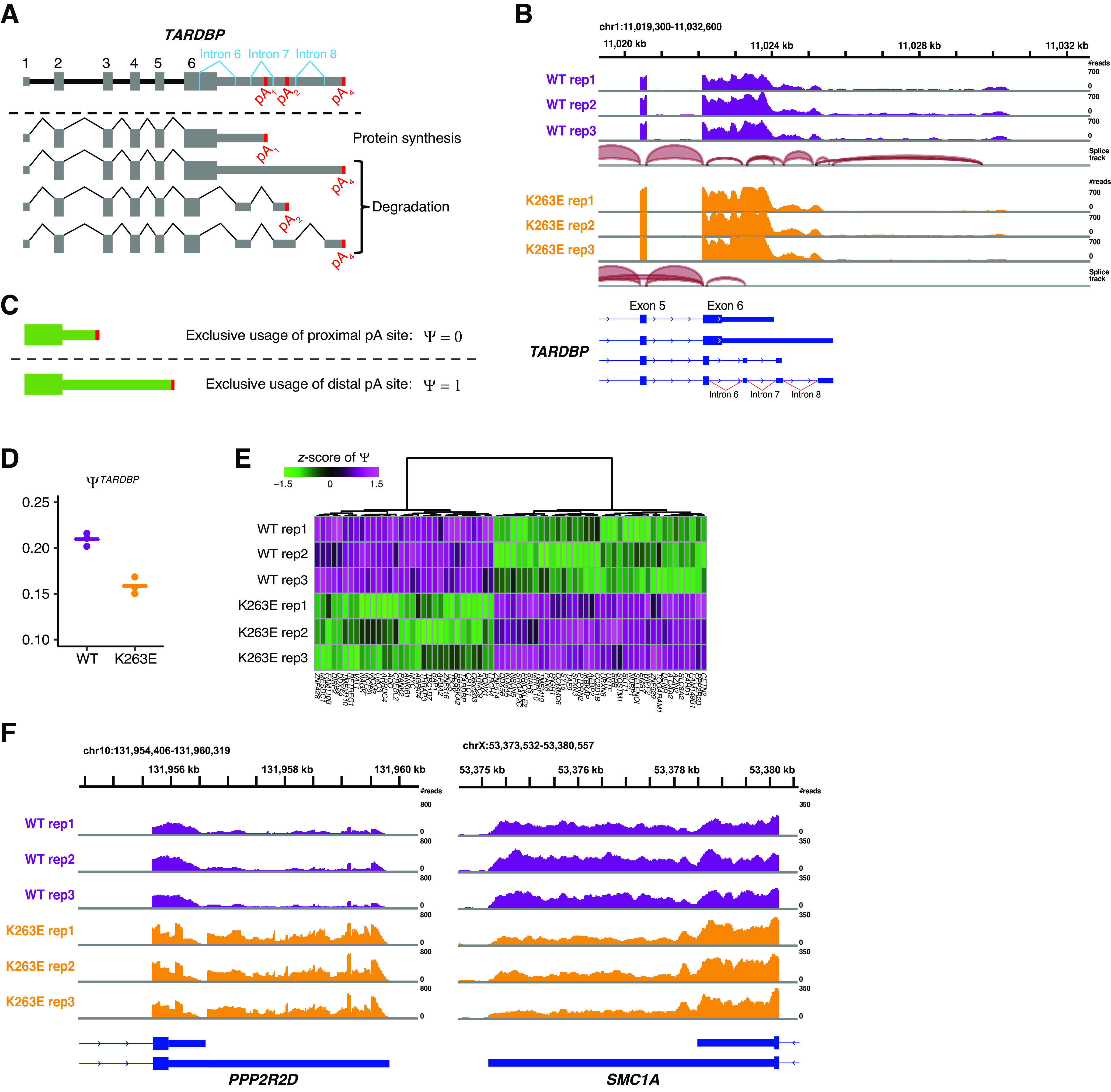
TDP-43^K263E^ impairs RNA 3′ end processing. ***A***, Schematic diagram of *TARDBP* mRNA isoforms. Alternative polyadenylation and splicing generate several isoforms, most of which are subject to degradation. ***B***, RNA-seq read coverage and splice junctions mapped to the genomic region of *TARDBP*. ***C***, Ψ as a metric of PAS choice. Genes that exclusively use the proximal PAS are assigned Ψ values of 0, whereas genes that exclusively use distal PAS are assigned Ψ values of 1. ***D***, Ψ values for *TARDBP* calculated from RNA-seq datasets in each of three replicates. ***E***, Heatmap showing genes with a significant change in Ψ based on a cutoff of 0.01 for *p* values. ***F***, RNA-seq read coverage mapped to the genomic regions of *PPP2R2D* and *SMC1A*. 3′ UTRs were differentially transcribed between wild-type and TDP-43^K263E^ neurons.

While gene expression profiling and splicing analyses indicated the similarity between TDP-43^K263E^ and TDP-43 knock-down, *TARDBP* expression was upregulated in TDP-43^K263E^ neurons (Fig. 1*D*). *TARDBP* expression was autoregulated by TDP-43 itself; namely, the binding of TDP-43 to its own mRNA changes the processing of the 3′ UTR, finally leading to a decrease in the mRNA level (Fig. 3*A*; Ayala et al., 2011; Eréndira Avendaño-Vázquez et al., 2012; Weskamp and Barmada, 2018). Indeed, we observed decreased splicing events in the 3′ UTR of the *TARDBP* mRNA in TDP-43^K263E^ neurons (Fig. 3*B*), suggesting that K263E mutations affect the autoregulatory properties of TDP-43. We focused on alternative polyadenylation, which results in the formation of multiple transcript isoforms with distinct 3′ UTRs, to further investigate 3′ UTR regulation. We quantified the usage level of each PAS from RNA-seq data (Ha et al., 2018), and gene-level PAS usage was summarized as the metric Ψ (Goering et al., 2021). Genes with exclusive usage of the most proximal PAS were assigned Ψ values of 0, whereas genes with exclusive usage of the most distal PAS were assigned Ψ values of 1 (Fig. 3*C*). The use of multiple PASs will result in Ψ values between 0 and 1, depending on the relative usage of individual sites. With this metric Ψ, we found that the 3′ UTR was shortened in TDP-43^K263E^ neurons compared with wild-type neurons (Fig. 3*D*). These results imply that the K263E mutation impairs the 3′ UTR regulation of the *TARDBP* mRNA, leading to the collapse of autoregulation.

### Global disruption of 3′ end processing

TDP-43 regulates not only its own mRNA but also a broad range of 3′ end processing events (Rot et al., 2017). Thus, we performed the differential PAS usage analysis with the metric Ψ and identified 70 genes whose PAS usage was significantly altered between wild-type and TDP-43^K263E^ neurons (Fig. 3*E*). The proximal PAS of *PPP2R2D* was preferentially used in wild-type neurons; on the other hand, TDP-43^K263E^ neurons used the lengthened 3′ UTR (Fig. 3*F*). The 3′ UTR of *SMC1A* was shifted similarly, but in the opposite direction (Fig. 3*F*). These shifts are consistent with a recent study that identified genes with PAS switches induced by TDP-43 depletion (Hallegger et al., 2021). Collectively, these results suggest that TDP-43^K263E^ affects RNA 3′ end processing of various genes because of the impaired TDP-43 function.

### The subcellular localization of TDP-43 is unaffected

**Figure 4.**
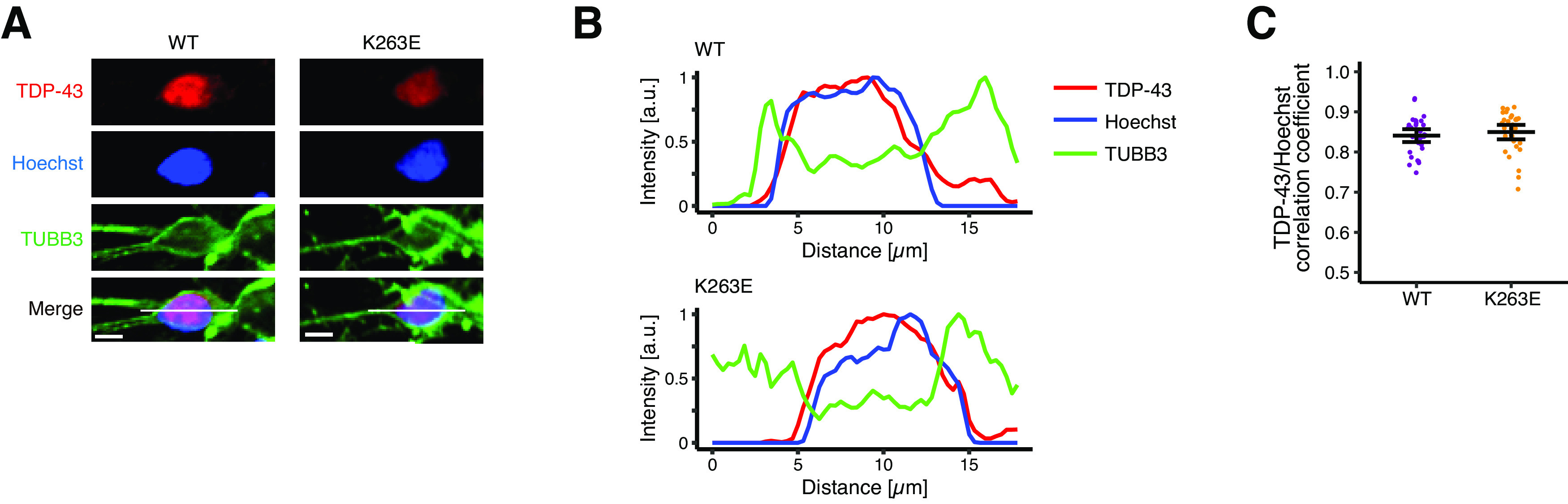
The subcellular localization of TDP-43 is not affected by the K263E mutation. ***A***, Representative images of TDP-43 in iPSC-derived cortical neurons. Scale bar: 5 μm. ***B***, Line-scan analysis along the solid white lines depicted in ***A***. ***C***, Pearson’s correlation coefficient for TDP-43 immunostaining and Hoechst signals (*n* = 32 cells from 4 independent experiments, mean with 95% confidence interval).

Based on our results, the LOF of TDP-43 has been suggested in TDP-43^K263E^ neurons. TDP-43 functions mainly in the nucleus, and its redistribution from the nucleus to the cytoplasm has been recognized as a pathologic hallmark of ALS and FTLD, suggesting that the pathogenic mechanism is associated with the loss of nuclear TDP-43 function (Mackenzie et al., 2010; G. Kim et al., 2020). Thus, we examined whether K263E exerted its LOF effect through subcellular mislocalization. In both wild-type and TDP-43^K263E^ neurons, we observed signals for TDP-43 immunostaining primarily in the nucleus (Fig. 4*A*,*B*). Pearson’s correlation coefficient revealed a strong correlation between TDP-43 immunostaining and the DNA counterstain in both groups (Fig. 4*C*). In addition, while intranuclear droplets of TDP-43 are suggested to be precursors of nuclear or cytoplasmic TDP-43 aggregates (Yu et al., 2021), TDP-43 was diffusely distributed within the nucleus in both wild-type and TDP-43^K263E^ neurons (Fig. 4*A*,*B*). Therefore, the TDP-43 subcellular or intranuclear localization was not altered by the K263E mutation.

### Cell type specificity of disrupted RNA processing induced by TDP-43^K263E^

**Figure 5.**
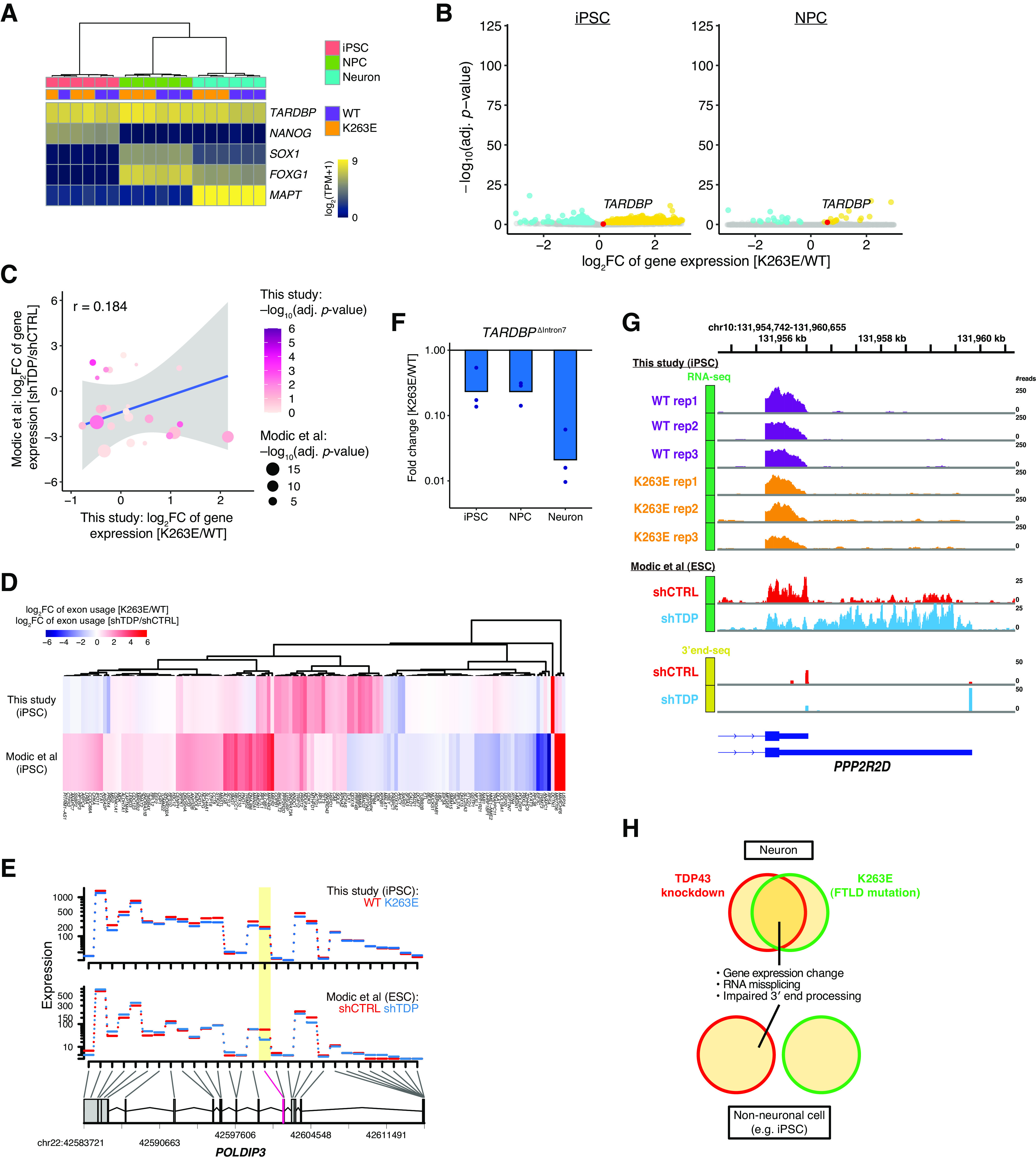
RNA processing impairments induced by TDP-43^K263E^ are not observed in nonneuronal cells. ***A***, Heatmap of gene expression in iPSCs, NPCs, and neurons. ***B***, Volcano plots showing genes with significantly altered expression between wild-type and TDP-43^K263E^ iPSCs and NPCs. ***C***, Scatter plot comparing fold changes in gene expression induced by TDP-43^K263E^ in iPSCs with those induced by TDP-43 knock-down in ESCs (Modic et al., 2019). DEGs in the study by Modic and colleagues (using a cutoff of 0.05 for Benjamini–Hochberg adjusted *p* values and a cutoff of 0.25 for the log2 fold change ratio) were analyzed. Shaded areas indicate 95% confidence intervals. Pearson’s correlation coefficients are shown. ***D***, Heatmap comparing fold changes in the exon usage induced by TDP-43^K263E^ in iPSCs with those induced by TDP-43 knock-down in ESCs. Differentially used exons in the study by Modic and colleagues (using a cutoff of 0.01 for Benjamini–Hochberg adjusted *p* values) were analyzed. ***E***, Visualization of the expression levels of each of the exons of *POLDIP3* in iPSC/ESC cultures. Exons with significantly different usage levels are highlighted in yellow. ***F***, Fold changes in the expression of the *TARDBP* isoform with intron 7 splicing in iPSC, NPC, and neuron cultures (normalized to *ACTB*; *n* = 3). ***G***, Top panel, RNA-seq read coverage mapped to the genomic region of *PPP2R2D* in wild-type and TDP-43^K263E^ iPSC cultures. Middle panel, RNA-seq read coverage in ESC cultures with TDP-43 knock-down. Bottom panel, 3′ end-seq coverage tracks in ESC cultures with TDP-43 knock-down. ***H***, Schematic illustrating neuron-specific effects of the K263E mutation on the function of TDP-43. In neuronal cell cultures, TDP-43^K263E^ phenocopies TDP-43 knock-down, including changes in gene expression, misregulation of RNA splicing, and aberrant 3′ end processing. On the other hand, in nonneuronal cell types, TDP-43^K263E^ does not exhibit LOF phenotypes.

We performed RNA-seq of iPSCs and NPCs expressing wild-type or K263E variant TDP-43 to test whether these RNA processing disruptions induced by TDP-43^K263E^ were also present in cell types other than neurons (Extended Data Fig. 1-1*A–E*). *TARDBP* was expressed at similarly high levels among iPSCs, NPCs, and neurons (Fig. 5*A*). In contrast to neurons, we did not find a drastic change of *TARDBP* expression in TDP-43^K263E^ iPSCs and NPCs compared with wild-type cells (Fig. 5*B*). We reanalyzed the RNA-seq data from ESCs with TDP-43 knock-down (Modic et al., 2019), and the comparison of the gene expression profiles showed no correlation between TDP-43^K263E^ iPSCs and TDP-43 knock-down ESCs (Fig. 5*C*), suggesting that the K263E mutation does not phenocopy TDP-43 knock-down in iPSC cultures. This dissimilarity between TDP-43^K263E^ iPSCs and TDP-43 knock-down ESCs was also evident in the differential exon usage analysis, in which exon usage fold changes induced by TDP-43^K263E^ were quite distinct from those induced by TDP-43 knock-down (Fig. 5*D*). Remarkably, the exclusion of *POLDIP3* exon 3 was only observed in TDP-43 knock-down ESCs but not in TDP-43^K263E^ iPSCs (Fig. 5*E*). Moreover, the K263E mutation drastically decreased intron 7 splicing in the 3′ UTR of *TARDBP* only in neurons; in contrast, this splicing event was rarely affected in TDP-43^K263E^ iPSCs and NPCs (Fig. 5*F*). In the analysis of the 3′ end processing of the *PPP2R2D* mRNA, RNA-seq and 3′ end-seq revealed that the 3′ UTR was lengthened in TDP-43 knock-down ESCs, but TDP-43^K263E^ iPSCs did not exhibit this PAS switch (Fig. 5*G*). Taken together, the data from nonneuronal cells, especially iPSCs, indicate that TDP-43^K263E^ did not mimic the RNA processing impairment induced by TDP-43 depletion. This result is the opposite of the close relationship between the K263E mutation and LOF in neurons (Fig. 5*H*).

## Discussion

Human iPSC-based disease models have remarkable potential to clarify disease pathogenesis and to discover effective therapies because of the discrepancy between patients and animal models (Okano and Yamanaka, 2014; Imaizumi and Okano, 2021). Previous studies of human iPSC-based models revealed RNA misprocessing induced by TDP-43 LOF; however, these studies largely used a knock-down strategy for recapitulating TDP-43 LOF (Klim et al., 2019; Prudencio et al., 2020), and the effect of pathogenic mutations of TDP-43 on global RNA regulation has not been extensively investigated. In the present study, we characterized global RNA processing impairments induced by the K263E mutation in iPSC-derived neurons. Consistent with the disrupted RNA binding capacity of TDP-43^K263E^ (H.J. Chen et al., 2019), substantial similarity exists between our mutant model and the knock-down system, indicating that pathogenic TDP-43 mutation induces TDP-43 LOF in neurons. Notably, our mutant model exhibited a defect in TDP-43 autoregulation, which cannot be investigated in a knock-down system.

Previous reports have suggested several distinct but overlapping effects of mutations on TDP-43 functions, including cytoplasmic mislocalization, an increased tendency to aggregate, and a decreased RNA binding capacity (Prasad et al., 2019). Cellular models overexpressing the TDP-43^K263E^ mutant indicate that K263E disrupts the RNA binding capacity of TDP-43 and enhances intranuclear TDP-43 aggregation or liquid shell formation (H.J. Chen et al., 2019; Yu et al., 2021). Although our results are consistent with the reduced RNA binding capacity of TDP-43^K263E^, we did not observe aggregation or droplets of TDP-43 in our iPSC-derived neurons. This inconsistency is probably because of the difference in TDP-43 expression levels. Excess TDP-43 expression might enhance TDP-43 aggregation, but endogenous TDP-43 expression does not. This finding is supported by a neuropathological study of a patient with FTLD carrying the K263E mutation, in which intranuclear TDP-43 inclusions were not observed in the cerebral cortex (Kovacs et al., 2009).

Additionally, cytoplasmic TDP-43 inclusions were indeed observed in this K263E carrier patient (Kovacs et al., 2009). In contrast to this pathologic observation, iPSC-derived neurons did not exhibit cytoplasmic mislocalization of TDP-43, consistent with previous reports using iPSC-derived neurons expressing other pathogenic TDP-43 mutations (Klim et al., 2019). On the other hand, cytoplasmic TDP-43 accumulation was observed in neurons directly converted from fibroblasts expressing another pathogenic TDP-43 mutation, N352S (Melamed et al., 2019). A recent study of Huntington’s disease suggested that age-related signatures were different between iPSC-derived neurons and directly converted neurons; therefore, pathogenic huntingtin aggregates were detected only in converted neurons but not in iPSC-derived neurons (Victor et al., 2018). This study revealed that the induction of pluripotency erased age marks, while direct neuronal conversion maintained age-related signatures. Therefore, direct neuronal conversion would be beneficial for investigating age-associated phenotypes associated with TDP-43 mutations reflecting more advanced pathology.

Our results strongly suggest the cell type-specific function of TDP-43^K263E^. TDP-43 LOF was only observed in neuronal cultures, whereas iPSCs and NPCs did not suffer from K263E mutation. The effect of TDP-43 mutation is often tested in nonneuronal cell lines, and comparisons among cell lines have been seldom studied. Thus, the findings for the TDP-43 mutant may need to be revisited in terms of the cell types tested. This cell type specificity is also important for the pathogenesis of ALS/FTLD. CNS-specific lesions in patients with ALS/FTLD do not match the observation that TDP-43 is expressed in various organs in the body (Sephton et al., 2010). Neuron-specific TDP-43 LOF may account for CNS-specific pathology. Although we do not yet know the mechanism of cell type-specific LOF, a neuron-specific cofactor of TDP-43 might have a role. Regardless, this study provides insights into the unprecedented cell type specificity of TDP-43 LOF and provides new opportunities for advancing ALS/FTLD research.

## Synthesis

Reviewing Editor: Karl Herrup, University of Pittsburgh School of Medicine Decisions are customarily a result of the Reviewing Editor and the peer reviewers coming together and discussing their recommendations until a consensus is reached. When revisions are invited, a fact-based synthesis statement explaining their decision and outlining what is needed to prepare a revision will be listed below. The following reviewer(s) agreed to reveal their identity: Sherif El-Khamisy, Fiona Song.

The manuscript represents an interesting analysis of TDP43 with the K263E mutation and its effect on RNA processing in differentiated neurons. They analyze the effects of a homozygous K263E mutation of TARDBP protein in human iPSCs. Using sequencing technology, they establish gene expression profiles from isogenic iPSCs harboring the TDP-43 K263E mutation and characterize the similarities and differences in splicing between wild type, knockdown, and mutant TDP43. They focus on the disruption of 3’ end processing and the cell type specificity of the TDP-43 mutation in inducing RNA processing disruption. The key findings are the effect of mutant TDP43 on 3’ RNA processing, TDP43 auto-regulation, alternative splicing, and highlight the disparity between progenitors and differentiated neurons. The manuscript deals with an interesting topic and lays the foundation for a fresh approach to an intriguing cellular protein. Though the manuscript has many strengths, the authors need to provide more solid evidence to prove their current conclusions and allow the work to realize its full potential.

The Results should be clarified as many of the data presented raise concerns about their interpretation. To begin, the authors should validate that the cells are behaving equivalently despite their genetics. Specifically, please provide evidence that neuronal induction was equivalent in all lines and specifically that the K263E cells were phenotypically similar to wild type.

With respect to the genes selected for the heatmap, the authors must clearly specify whether all the genes that overlap between the two datasets are included or have the data been further filtered (e.g. by expression). The number of genes seems quite small when compared to the number of differentially expressed genes in each experiment. If this is just because the overlap between the two datasets was small, then this should be explicitly stated. If this is not the case, the small number of genes is a serious concern. The authors called the differentially expressed genes from the RNA seq data with a cutoff of 0.01 for Benjamini-Hochberg adjusted p values and a cutoff of 0.5 for the log2 fold change ratio. Using 0.5 as the cutoff of log2 fold change ratio is barely significant (fold change = 1.14). However, the authors only identified 233 DEGs from the data which indicates the quality of the data is not ideal. They should either address this issue or find a way to justify the result. As presented, the data raise concerns about the quality of the sequencing data. From a methodological standpoint, the authors need to inform the reader what quality control was performed on the RNA seq reads, as well as which transcript index and version was used (e.g. ensemble, refseq etc)? Also, please indicate whether in the alternative polyadenylation analysis the PAS positions were the same as in the original QAPA publication or instead came from an updated build/ genome model.

Only the data from iPSC-derived cortical neurons showed very few significant differential genes. Other sequencing data did not reach the cutoff. This is concerning. They author should include more details of their data processing and results of quality control to convince the readers that their negative results are not from artificial errors of the experiments.

The annotation-free differential splicing analysis is intriguing. In order to make the data more convincing, the author should show their strategy on selecting PFKP and DNAJC5. Why just pick these two? Are they the ones with lowest p-value?

In figure 1G there are three scales for adjusted p-values. The scales from the two publicly available datasets are arranged low to high but the scale from the novel data is arranged high to low. Please change this so all scales go in the same direction.

From the data in figure 2E, it appears that there were only 3 unannotated intron splicing events detected. A summary of all events would be helpful to put the three genes chosen by the authors into context. In addition, since submission of this manuscript, an analysis of TDP43 loss in ALS/FTD has been published (https://doi.org/10.1038/s41586-022-04424-7). This provides a mechanism for reduced UNC13A expression. The authors should highlight this gene in differential exon usage plots and exon expression level plots to further validate that their model fits with this newly published data.

In Figure 3F the IGV tracks appear to ‘top out’ in the K263E (orange) tracks. This could mask true maximum values and distort the comparisons with WT cells. The scale should be adjusted so the full range of values can be seen whilst maintaining the same scale maxims and minima for all conditions.

The expression level in the current data is approximately two times the level reported by Modic et al. Please address this issue and explain the reason(s) for the difference in expression level between the two datasets and whether this might have affected differential exon usage.

Please clarify whether the Venn diagrams shown in Figure 5F are based on data or intended simply as an illustration. If the findings are based on real data then more details are needed for the reader to be fully informed as to their significance.

Finally, please ensure that the series numbers in Gene Expression Omnibus are working properly.

## Author Response

Imaizumi et al.

Point-by-point responses to the reviewers’ comments

We thank the reviewers for evaluating our manuscript entitled “Pathogenic mutation of TDP-43 impairs RNA processing in a cell type-specific manner: implications for the pathogenesis of ALS/FTLD” (eN-NWR-0061-22).

We found the comments made by the reviewers to be very helpful, and we have addressed the comments as thoroughly as possible. Below, the reviewers’ comments are presented in bold, and our responses are printed below. The changes made to the revised manuscript are highlighted in red.

1. To begin, the authors should validate that the cells are behaving equivalently despite their genetics. Specifically, please provide evidence that neuronal induction was equivalent in all lines and specifically that the K263E cells were phenotypically similar to wild type.

> We confirmed the equivalence of cellular identity between wild-type and TDP-43K263E cells by the following steps. First, we confirmed that TDP-43K263E iPSCs had a similar morphology to wild-type iPSCs (Fig. 1C). We also examined the expression of marker genes for pluripotency, neural progenitors, and neurons by RT-qPCR, and there was no difference in these marker gene expressions between wild-type and TDP-43K263E cells (Fig. 1E). Finally, both wild-type and TDP-43K263E iPSCs had a similar neuronal induction efficiency (Fig. 1F). These data indicate that the mutant TDP-43 did not affect the maintenance of pluripotency and neuronal induction.

These observations are now presented in Figures 1C, E, and F, and we included the following statements summarizing these results.

(page 7, line 190-194)

This mutant genotype did not affect the gene expression of markers for pluripotency, neural progenitors, and neurons (Fig. 1E). In addition, we found no difference in neuronal induction efficiency (Fig. 1F). These data indicate that the pluripotency maintenance and the neuronal induction were equivalent between wild- type and TDP-43K263E iPSCs.

2. With respect to the genes selected for the heatmap, the authors must clearly specify whether all the genes that overlap between the two datasets are included or have the data been further filtered (e.g. by expression). The number of genes seems quite small when compared to the number of differentially expressed genes in each experiment. If this is just because the overlap between the two datasets was small, then this should be explicitly stated. If this is not the case, the small number of genes is a serious concern. The authors called the differentially expressed genes from the RNA seq data with a cutoff of 0.01 for Benjamini-Hochberg adjusted p values and a cutoff of 0.5 for the log2 fold change ratio. Using 0.5 as the cutoff of log2 fold change ratio is barely significant (fold change = 1.14). However, the authors only identified 233 DEGs from the data which indicates the quality of the data is not ideal. They should either address this issue or find a way to justify the result. As presented, the data raise concerns about the quality of the sequencing data. From a methodological standpoint, the authors need to inform the reader what quality control was performed on the RNA seq reads, as well as which transcript index and version was used (e.g. ensemble, refseq etc)? Also, please indicate whether in the alternative polyadenylation analysis the PAS positions were the same as in the original QAPA publication or instead came from an updated build/ genome model.

> We firstly evaluated the quality of our RNA-seq data, and all samples were of good quality. Next, we assessed the number of DEGs. Finally, we summarized the information regarding the genome build and index used in this study. The details of these analyses are described below in three parts.

1. Quality control of RNA-seq samples

The RNA quality was evaluated by RNA integrity number (RIN), and all samples were kept with > 9 (Fig. S1A). The average Phred quality score of sequencing reads was > 30, representing 99.9% accuracy (Fig. S1B). Further quality filtering by fastp removed only < 2% of reads (Fig. S1C). There was no difference in the mapping rate of alignment by HISAT2 and pseudo-alignment by Salmon among all sequencing samples (Fig. S1D and E). Thus, our quality control procedures assured that the quality of RNA-seq data was satisfactory.

These quality control data are included in Figures S1A-E, and we added the description as follows.

2. DEG analysis

In Figure 1G (previous Figure 1D), there were a relatively small number of DEGs (233 genes) identified with a cutoff of 0.01 for adjusted p values and a cutoff of 0.5 for the log2 fold change ratio. However, there was no problem with the quality of RNA-seq data as described above, and this small number is not due to the RNA- seq quality. Rather, this low number indicates that TDP-43 loss-of-function affects the expression of a distinct subset of genes. This is also supported by the fact that the DEG analysis of the dataset from Klim et al. (2019) also identified a limited number of genes (78 genes) with the same thresholds.

In Figures 1J-K (previous Figures 1F-H), we selected 42 genes as intersections between the DEG lists from studies by Klim et al. (2019) and Melamed et al. (2019) and we did not set additional filters to narrow down this geneset. The small number of genes in this geneset is not only due to the small number of DEGs, but also to the different cell types used in these two studies, because Klim et al. (2019) and Melamed et al. (2019) used iPSC-derived neurons and SH-SY5Y cells, respectively.

To test whether this small number of genes biases the results in Figures 1J-K, we changed the cutoff criteria of DEG analyses. We newly set a cutoff of 0.05 for adjusted p values and a cutoff of 0.25 for the log2 fold change ratio. These cutoffs identified 73 genes as intersections between the DEG lists from studies by Klim et al. and Melamed et al. PCA and fold change comparisons based on these genes showed similar results to the previous analyses using a smaller number of DEGs (Figures 1J-K). These results indicate that a small number of genes analyzed does not cause a bias in PCA and fold change comparisons.

Collectively, we updated Figures 1G-K with the new cutoff criteria (adjusted p values < 0.05, log2 fold change > 0.25) as shown below.

For consistency, we also updated Figures 5B and C with the new cutoff criteria as follows. This change did not affect the overall results.

3. Genome build and index used in the study In gene expression quantification by Salmon, we used the GENCODE annotation (release 33, version GRCh38). In the differential exon usage analysis, sequencing reads were aligned to the human GRCh38 genome by HISAT2, and counts at individual exons were quantified at individual exons by featureCounts with Ensembl annotation (release 104).

While the original QAPA paper utilized GRCh37 genome annotation (Ha et al., 2018), we used the GRCh38- compatible PAS annotations available on the QAPA GitHub page (https://github.com/morrislab/qapa).

We included the information regarding the genome build and index used in these analyses as follows.

(page 4, line 108-109)

Salmon (Patro et al., 2017) was used to generate the TPM using the transcript index from the reference GRCh38 genome annotation (GENCODE release 33) ...

(page 4, line 119-120)

Counts at individual exons were calculated from the HISAT2-aligned data using featureCounts (Liao et al., 2014) with the gene annotation from Ensembl (release 104) ...

(page 4, line 133-135)

3! UTR sequence was extracted from GRCh38 genome by “qapa fasta# based on the pre-complied annotation available on the QAPA GitHub page (https://github.com/morrislab/qapa).

3. Only the data from iPSC-derived cortical neurons showed very few significant differential genes. Other sequencing data did not reach the cutoff. This is concerning. They author should include more details of their data processing and results of quality control to convince the readers that their negative results are not from artificial errors of the experiments.

> As described above in comment #2, there are no problems with the quality of our RNA-seq data. It should be also noted that markers for pluripotency and neural progenitors were robustly detected, ensuring appropriate quality of RNA-seq data (Figure 5A). These results suggest that few DEGs in iPSCs and NPCs did not result from low quality of sequencing, but rather, there is indeed a little difference in gene expression between wild-type and TDP-43K263E iPSCs/NPCs.

4. The annotation-free differential splicing analysis is intriguing. In order to make the data more convincing, the author should show their strategy on selecting PFKP and DNAJC5. Why just pick these two? Are they the ones with lowest p-value?

> We picked up these three genes because cryptic splicing of these genes has been reported in TDP-43 knockdown models (Polymenidou et al., 2011; Klim et al., 2019). Meanwhile, after the initial submission of this manuscript, two papers reported that the splicing patterns of some novel target genes are affected by TDP-43 loss (Brown et al., 2022; Ma et al., 2022). We reviewed our annotation-free differential splicing analysis in detail and detected missplicing of these target genes, including UNC13B, KALRN, and SYT7. Thus, in addition to focusing on three genes, we summarized the annotation-free differential splicing analysis as follows.

(page 8, line 226-229)

We detected cryptic splicing not only in STMN2 but also in various gene targets, including PFKP, DNAJC5, KALRN, SYT7, and UNC13B, all of which have been shown to be misspliced in TDP-43 knockdown models (Fig. 2E, F) (Polymenidou et al., 2011; Klim et al., 2019; Brown et al., 2022; Ma et al., 2022).

Please see also comment #6, especially regarding the evaluation of UNC13A splining, which is one of the main findings of these two papers (Brown et al., 2022; Ma et al., 2022).

5. In figure 1G there are three scales for adjusted p-values. The scales from the two publicly available datasets are arranged low to high but the scale from the novel data is arranged high to low. Please change this so all scales go in the same direction.

> We revised Figure 1J (previous Figure 1G) and 5C as the reviewers suggested.

6. From the data in figure 2E, it appears that there were only 3 unannotated intron splicing events detected. A summary of all events would be helpful to put the three genes chosen by the authors into context. In addition, since submission of this manuscript, an analysis of TDP43 loss in ALS/FTD has been published (https://doi.org/10.1038/s41586-022-04424-7). This provides a mechanism for reduced UNC13A expression. The authors should highlight this gene in differential exon usage plots and exon expression level plots to further validate that their model fits with this newly published data.

> As the reviewers suggested, we added a summary of the annotation-free splicing analysis in Figure 2E. In addition to three genes, we detected a significant number of genes that have been shown to be misspliced by TDP-43 loss in previous reports. Specifically, these genes include KALRN and SYT7, whose cryptic splicing has been reported in the paper that the reviewers mentioned. Please see also comment #4.

However, we failed to detect significant cryptic splicing events in UNC13A in the annotation-free differential splicing analysis. To eliminate the possibility that our parameter setting might affect the detection sensitivity, we firstly reran LeafCutter annotation-free splicing analysis with a new parameter setting with reference to previous reports (Brown et al., 2022; Ma et al., 2022), but UNC13A splicing was still not detected. To further evaluate UNC13A splicing, we extracted sequencing reads spanning the cryptic exon from our RNA-seq data. While there were no reads that span the cryptic exon in wild-type neurons, we detected very few spanning reads in two of three samples of TDP-43K263E neurons (Figures S2A-C). A similar analysis targeting STMN2 cryptic exon robustly detected splicing change by mutant TDP-43 (Figures S2D-F). These results raised two possibilities. One possibility is that TDP-43K263E has little if any effect on UNC13A splicing; and the second is that RNA sequencing could not well capture UNC13A cryptic exons in spite of the fact that missplicing occurs. We found that wild-type and isogenic TDP-43K263E cells harbored homozygous risk SNP/indels that increase the efficiency of UNC13A cryptic splicing upon TDP-43 loss (Figure S2G) (Brown et al., 2022; Ma et al., 2022). Thus, the cryptic splicing of UNC13A in these cells is expected to be drastically enhanced by TDP-43 LOF, which supports the second possibility. Indeed, RT-qPCR reproducibly detected UNC13A cryptic splicing in TDP-43K263E neurons (Figure S2H). These data suggest that UNC13A missplicing was actually occurring, but RNA-seq analysis was not sensitive enough to detect it. The low sensitivity of RNA-seq analysis to detect UNC13A missplicing was also supported by the finding that analyses using the data from Klim et al. (2019) and Melamed et al. (2019) similarly detected UNC13A missplicing in only a subset of samples (Figure S2I).

These data are now presented in Figures S2A-I, and we included the following statements.

(page 8, line 229-246)

On the other hand, this annotation-free splicing analysis could not detect UNC13A cryptic splicing, which has recently been reported as a hallmark of TDP-43 LOF (Brown et al., 2022; Ma et al., 2022). To further evaluate UNC13A splicing, sequencing reads spanning the cryptic exon were extracted and quantified. We detected very few spanning reads in only a subset of samples of TDP-43K263E neurons, whereas there were no spanning reads in wild-type neurons (Figures S2A-C). A similar analysis focusing on STMN2 cryptic exon robustly detected splicing change by mutant TDP-43 (Figures S2D-F). These results raised two possibilities: one is that TDP-43K263E has little if any effect on UNC13A splicing; and the other is that RNA sequencing could not well capture UNC13A cryptic exons in spite of the fact that missplicing occurs. We found that both wild-type and isogenic TDP-43K263E cells harbored homozygous risk SNP/indels (rs12973192, rs12608932, and rs56041637) that increase the efficiency of UNC13A cryptic splicing upon TDP-43 loss (Figure S2G) (Brown et al., 2022; Ma et al., 2022). Thus, the cryptic splicing of UNC13A in these cells is expected to be drastically enhanced by TDP-43 LOF, which supports the second possibility. Indeed, RT- qPCR analysis revealed that UNC13A cryptic splicing was reproducibly increased in TDP-43K263E neurons (Figure S2H). These data suggest that UNC13A missplicing was actually occurring, but RNA-seq analysis was not sensitive enough to detect it. The low sensitivity of RNA-seq analysis to detect UNC13A missplicing was also supported by the finding that analyses using the data from Klim et al. (2019) and Melamed et al. (2019) similarly detected UNC13A missplicing in only a subset of samples (Figure S2I).

We also updated the parameter setting used in LeafCutter analysis as described below. (page 4, line 122-127)

Differentially spliced intron clusters were analyzed from the HISAT2-aligned data without existing isoform annotations by LeafCutter (Li et al., 2018). Briefly, splice junction reads were extracted with RegTools (Cotto et al., 2021) using a minimum of 6 bp as an anchor on each side of the junction. Junctions from each sample were then clustered using ‘leafcutter_cluster_regtools_py3.py’ (minclureads = 10). Differential intron splicing was calculated using ‘leafcutter_ds.R’.

7. In Figure 3F the IGV tracks appear to ‘top out’ in the K263E (orange) tracks. This could mask true maximum values and distort the comparisons with WT cells. The scale should be adjusted so the full range of values can be seen whilst maintaining the same scale maxims and minima for all conditions.

> We revised Figure 3F so that the scales were adjusted as follows.

8. The expression level in the current data is approximately two times the level reported by Modic et al. Please address this issue and explain the reason(s) for the difference in expression level between the two datasets and whether this might have affected differential exon usage.

> The RNA-seq data from Modic et al. (2019) were derived from total RNA without polyA selection, which contains not only mRNA, but also non-coding RNA. Thus, it is difficult to directly compare the gene expression levels with those of our data from polyA-based mRNA selection. We circumvented this problem by comparing fold changes of gene expression and exon usage in the two datasets, rather than directly comparing expression levels.

9. Please clarify whether the Venn diagrams shown in Figure 5F are based on data or intended simply as an illustration. If the findings are based on real data then more details are needed for the reader to be fully informed as to their significance.

> Figure 5F is not based on the actual data, but simply summarizes the neuron-specific effects of the K263E mutation as an illustration. We added the following description in the legend of Figure 5F.

H, Schematic illustrating neuron-specific effects of the K263E mutation on the function of TDP-43.

10. Finally, please ensure that the series numbers in Gene Expression Omnibus are working properly.

> The status of our GEO submissions was inadvertently set to private. It has now been changed to public and everyone can access the raw data.
